# A Protocol for Surgical Preparation of Rats for In Vivo Fiber Optic Photometry Using Genetically Encoded Fluorescent Biosensors

**DOI:** 10.3390/mps9050144

**Published:** 2026-10-02

**Authors:** Margarita A. Khlystova, Andrei M. Karhov, Veronika A. Katrukha, Maxim A. Solotenkov, Vladimir A. Mitkevich, Alexander A. Makarov, Vsevolod V. Belousov, Andrei B. Fedotov, Ilya V. Fedotov, Dmitry S. Bilan

**Affiliations:** 1 M.M. Shemyakin and Yu.A. Ovchinnikov Institute of Bioorganic Chemistry, Russian Academy of Sciences, Moscow 117997, Russia; margaritakhlystova119@gmail.com (M.A.K.); akarchoff@gmail.com (A.M.K.); katrukhav@gmail.com (V.A.K.); belousov@fccps.ru (V.V.B.); 2 Federal Center of Brain Research and Neurotechnologies, Federal Medical Biological Agency, Moscow 117513, Russia; 3 Faculty of Physics, M.V. Lomonosov Moscow State University, Moscow 119991, Russia; maksolo@list.ru (M.A.S.); a.b.fedotov@physics.msu.ru (A.B.F.); 4 Engelhardt Institute of Molecular Biology, Russian Academy of Sciences, Moscow 119991, Russia; mitkevich@gmail.com (V.A.M.); aamakarov@eimb.ru (A.A.M.); 5 Institute of Translational Medicine, Pirogov Russian National Research Medical University, Ministry of Health of the Russian Federation, Moscow 117513, Russia

**Keywords:** fiber-optic photometry, fiber implantation, in vivo recording of fluorescence signals, genetically encoded fluorescent biosensor, adeno-associated virus

## Abstract

Genetically encoded biosensors based on fluorescent proteins in combination with fiber-optic neurointerface technology constitute a powerful tool for neurobiological research. Optical fibers can be implanted into the target brain regions of laboratory mammals, while the genetically encoded nature of biosensors enables in vivo monitoring of biochemical processes at the level of specific cell types and their compartments. As the global collection of biosensors for various biological parameters continues to expand, this approach is gaining increasing popularity. The ability to record fluorescent signals in real time, as well as at intervals over extended periods of several weeks or even months, makes the method broadly applicable for studying both acute and chronic processes in animal models of neurological disorders. Here, we describe in detail the protocol for preparing rats for fiber-photometry experiments using implantation of three optical fibers as an example. The protocol provides detailed instructions for the fabrication of universal optical probes that are compatible with both commercially available fiber-photometry systems and custom optical setups.

## 1. Introduction

Genetically encoded biosensors based on fluorescent proteins are finding increasing use in modern neurobiological research. Most biosensors are chimeric protein molecules composed of several domains. The sensory domain is responsible for interaction with a specific cellular parameter, which can be a specific metabolite or a signaling molecule. The reporter domain is represented by a fluorescent protein, one or two in the case of biosensors based on Fluorescence Resonance Energy Transfer (FRET). The fluorescence signal reflects the state of the sensory part (its ligand-bound or free forms, oxidized or reduced states, phosphorylated or dephosphorylated forms, etc.). Thus, the dynamics of the fluorescence in real time reflect the dynamics of the biological parameter being studied. To date, the global collection of genetically encoded biosensors is represented by a wide variety of probes with diverse spectral and biochemical characteristics [1,2]. Genetically encoded fluorescent biosensors have become widely used in the redox biology field. Molecular tools of this type have enabled visualization of biological processes involving highly reactive and therefore short-lived intracellular chemical compounds, including hydrogen peroxide [3], hypohalous acids [4], and nitric oxide [5]. Redox biosensors are widely used in biological systems of varying complexity, and this is their key advantage [6]. Selecting cell-type-specific promoters enables targeted expression of biosensors in selected cells, whereas adding signal peptide tags allows their localization to a desired intracellular compartment.

The broad adoption of genetically encoded fluorescent biosensors has been driven by advances in the development and optimization of technologies for detection of a fluorescence signal in living systems. In neurobiology, neuronal cell cultures and ex vivo brain tissue preparations are the most accessible experimental models for studies using fluorescent biosensors and commercially available imaging platforms (confocal and widefield microscopy). However, understanding most complex biological processes, both under physiological conditions and during the development of pathologies, requires in vivo models. For example, acute (ischemic stroke, traumatic injury) or chronic (neurodegenerative diseases) pathological conditions of the brain involve complex interactions among different cell types and tissues that are difficult or, in some cases even impossible to simulate in vitro. The diversity of approaches for modeling brain pathologies in rodents, with the extensive collection of transgenic animal lines available worldwide makes in vivo studies the most promising, both for understanding the mechanisms underlying neurological disorders and for translation research aimed at the screening and testing of novel therapeutic strategies. The genetically encoded tools substantially expanded the range of experimental approaches available to researchers. Genes encoding such molecular tools can be delivered to the tissues of laboratory animals using viral gene delivery systems. In particular, different serotypes of adeno-associated viruses (AAVs) are widely used for this purpose [7,8]. Viral particles are injected directly into the target brain regions of anesthetized animals. Following injection of AAVs, about 3–5 weeks are typically required for the development of a pronounced fluorescent signal in the transduced tissues. In vivo detection of the signal can be performed through cranial windows using fluorescence microscopy, including the multiphoton excitation mode, which significantly improves spatial resolution at greater tissue depths [9,10,11]. Approaches to the miniaturization of head-mounted microscopes (miniscopes) are being actively developed. Such portable systems in different variations are now available for both one-photon and multiphoton fluorescence imaging modes [12,13,14,15]. However, these approaches are limited to measurements in superficial brain tissues, primarily within the cerebral cortex of laboratory animals. An alternative approach is based on the use of a fiber-optic neurointerface technology. Fluorescence-exciting light is transmitted through an optical fiber implanted into the tissue, and the emitted fluorescence is collected through the same fiber in the opposite direction. In this case, the integrated fluorescence signal is detected from the brain tissue surrounding the distal tip of the implanted fiber. The key advantage of this technique is the ability to record a fluorescence signal in vivo at virtually any coordinate of interest in the animal brain [16,17,18,19,20,21].

In our previous studies [22,23,24,25,26], we investigated in vivo dynamics of redox processes in the brain tissue of rats during the development of ischemic stroke in a widely used model of middle cerebral artery occlusion. Measurements of signals can be performed both in real time during the acute phase of pathogenesis and at intervals over several days after ischemia/reperfusion. Signal monitoring can be performed simultaneously in several regions of the brain by implanting multiple optical fibers. In our studies, we used different optical configurations to record fluorescence signals in brain cells expressing cpYFP-based biosensors HyPer7 [3] and SypHer3s [27]. We used custom-designed implantable optical probes that are compatible with both commercially available fiber-optic photometry systems and custom optical setups. The adapter part of the probe mounted on the skull surface has a universal design and is suitable for both rats and mice, while the length of the implanted fiber can be changed. In most publications, including ours, the main emphasis is focused on the biological findings, while the technical aspects are typically described briefly in the Materials and Methods sections. Here, we present a detailed protocol for the fabrication of optical probes, their quality tests, intracranial injection of AAV suspension into rat brain tissue for the delivery of a biosensor gene, and subsequent implantation of fibers at three coordinates during the same surgical procedure.

## 2. Experimental Design

**Figure 1:**
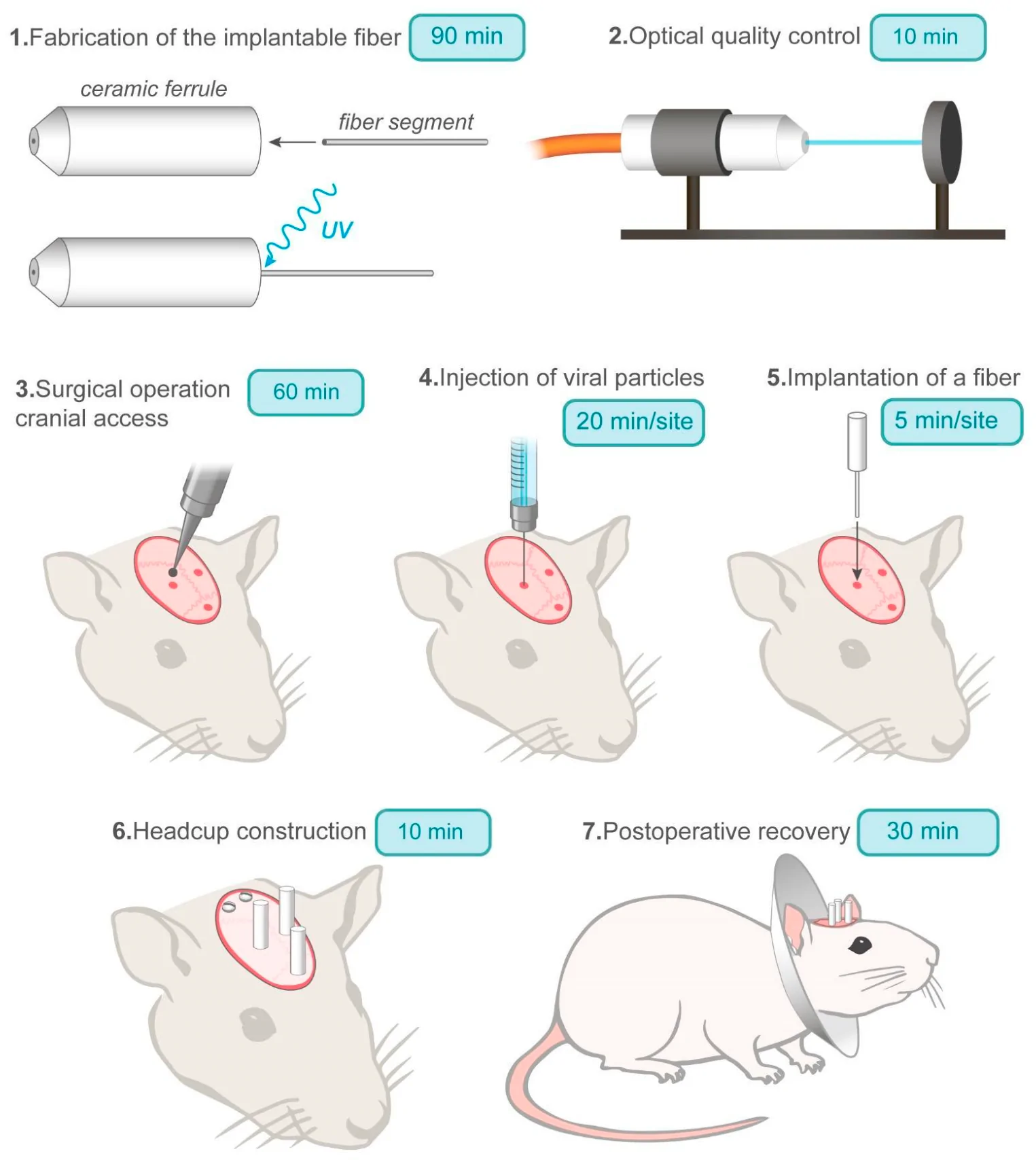
The general scheme of the protocol.

The protocol described here provides step-by-step procedures for preparing rats (*Rattus norvegicus*) for fiber-optic photometry experiments that enable in vivo recording of the fluorescent signals of genetically encoded biosensors in deep brain regions. We use this protocol to prepare animals for in vivo studies of redox dynamics and acidosis in brain tissue during ischemic stroke development caused by occlusion of the middle cerebral artery (MCAO), which is widely regarded as the most frequently used experimental model [28]. However, the protocol is universal and can be readily adapted to other models in combination with any fluorescent biosensors. The number of implanted optical fibers, as well as the brain coordinates, can be tailored to the specific objectives of each study. Animal preparation (described here using rats as an example, although the protocol is applicable to mice) consists of the following stages: (1) the fabrication of optical implants; (2) their quality control; (3) surgical field preparation; (4) stereotaxic injection of viral particles with the gene of the desired biosensor into brain tissue; (5) implantation of the optical fiber at the same coordinates as the injection site; (6) secure and well tolerated by the animal fixation of the implant connector to the skull; and (7) a postoperative recovery period. After such an operation, experiments can be performed after 2–3 weeks, depending on the level of expression and brightness of the biosensors used. The general scheme of the protocol can be found in Figure 1.

### 2.1. Materials

1. Ketoprofen (VIC Animal Health, Vitebsk, Belarus, Cat. no.: N/A).
2. Bupivacaine (PJSC Ozon Pharmaceuticals, Zhigulievsk, Russia, Cat. no.: N/A).
3. Insulin syringes (1 mL, fine needle) (MedCare, Foshan, China, Cat. no.: N/A).
4. Light-curing flowable composite (VladMiVa, Belgorod, Russia, Cat. no.: N/A).
5. 0.9% NaCl sterile saline (Bionit, Vladimir, Russia, Cat. no.: N/A).
6. Hydrogen peroxide solution (Ruskern, Moscow, Russia, Cat. no.: N/A).
7. 70% ethanol (Ecolab, Elektrogorsk, Russia, Cat. no.: N/A).
8. Ophthalmic ointment (Vita-POS) (URSAPHARM Arzneimittel GmbH, Saarbrücken, Germany, Cat. no.: N/A).
9. Hemostatic agent (AlCl_3_- or FeSO_4_-based) (Omega Dent, Moscow, Russia, Cat. no.: N/A).
10. Sodium hypochlorite solution (5–15% available chlorine) (Neva, St. Petersburg, Russia, Cat. no.: N/A).
11. Dental cement (powder + liquid components) (Yamahachi, Gamagori, Japan, 23B1X10001C03002).
12. Anchoring bone screw (Stoelting Co, Wood Dale, IL, USA, Cat. no.: 51457).
13. Tissue adhesive (e.g., Vetbond) (3M, St. Paul, MN, USA, Cat. no.: 1469SB).
14. Povidone–iodine (Betadine) (EGIS Pharmaceuticals PLC, Budapest, Hungary).
15. Parafilm (Bemis, Neenah, WI, USA, Cat. no.: PM996).
16. PVC tablecloth for collar (HOLAROOM, Yiwu, China, Cat. no.: TO76B).
17. Step-index multimode optical fiber, 105/125 µm core/cladding (Thorlabs, Newton, NJ, USA, Cat. no.: FG105UCA).
18. UV-curable optical adhesive (Norland Optical Adhesive 61, Norland Products, Inc., Cranbury, NJ, USA, Cat. no.: NOA61).
19. LC/PC-compatible ceramic ferrules (Technomash, Moscow, Russia, Cat. no.: НОК-ОМ-1.2).
20. Ceramic LC mating sleeves (Thorlabs, Newton, NJ, USA, Cat. no.: ADAL1).
21. Double-sided adhesive tape (3M, St. Paul, MN, USA, Cat. no.: 7100369955).
22. Lint-free optical wipes (Thorlabs, Newton, NJ, USA, Cat. no.: KW32).
23. Protective furcation tubing (Thorlabs, Newton, NJ, USA, Cat. no.: FT900KK).
24. Adhesive heat-shrink tubing (3M, St. Paul, MN, USA, Cat. no.: 7000099086).
25. Polishing films (Thorlabs, Newton, NJ, USA, Cat. no.: CK03).
26. Isopropyl alcohol (High Chemical New Materials Co Ltd., China, Shandong, Cat. no.: 29051200).

### 2.2. Equipment

- Stereotaxic frame with digital readout (Stoelting Co, Wood Dale, IL, USA, Cat. no.: 51500D).
- Isoflurane anesthesia machine VetFlo-0730 (vaporizer + induction chamber) (Kent Scientific, Torrington, CT, USA, Cat. no.: 78100-00).
- Hamilton syringe 10 µL (microliter syringe) (Hamilton Company, Bonaduz, Switzerland, Cat. no.: 7635-01).
- Microinjection pump/injector controller (Stoelting Co, Wood Dale, IL, USA, Cat. no.: 53311).
- Drill bits, flat tip (or round-tip) 0.5 mm (RWD LifeScience In, Shenzhen, China, Cat. no.: 78040).
- Wide trapezoidal drill bits (for anchoring screw hole) (RWD LifeScience In, Shenzhen, China, HM31010, Cat. no.: 78061).
- Surgical drill (RWD LifeScience In, Shenzhen, China, Cat. no.: 78001).
- Scalpel (Stoelting Co, Wood Dale, IL, USA, Cat. no.: 52171P).
- Curved-tip tweezers (Stoelting Co, Wood Dale, IL, USA, Cat. no.: 52102-36P).
- Surgical scissors (Stoelting Co, Wood Dale, IL, USA, Cat. no.: 52132-06P).
- Double-ended cement spatula (Sammar International, Sialkot, Pakistan, Cat. no.: 112-TNPFI8A).
- Optical-fiber ferrules (Technomash, Moscow, Russia, Cat. no.: НОК-ОМ-1.2).
- Cannula holder (stereotaxic attachment) (Thorlabs, Newton, MA, USA, Cat. no.: XCL).
- Rectal thermometer probe (Kent Scientific, Torrington, CT, USA, Cat. no.: RT-0001-9).
- UV-curing light (Foshan Niceden Medical Equipment Co., Foshan, China, Cat. no.: CU-10201).
- Optical-fiber stripper for fibers with a 125 µm cladding diameter (Thorlabs, Newton, NJ, USA, Cat. no.: T06S13).
- Fiber cleaver (Thorlabs, Newton, NJ, USA, Cat. no.: XL411).
- Computer-controlled motorized translation stage (Thorlabs, Newton, NJ, USA, Cat. no.: MT1/M-Z8).
- Custom-built fiber-cleavage frame.
- Optical or stereoscopic microscope for fiber assembly and endface inspection (MCscope, Wuhan, China, Cat. no.: MC-TZG5X-AF200).
- UV-curing source suitable for NOA61 (DarkBeam, Shenzhen, China, Cat. no.: V5-DB-395NM-2).
- 470 nm light source with suitable optics for coupling light into the connecting patch cable (Thorlabs, Newton, NJ, USA, Cat. no.: M470F4).
- Optical power meter (Thorlabs, Newton, NJ, USA, Cat. no.: PM100D).
- Photodiode power sensor (Thorlabs, Newton, NJ, USA, Cat. no.: S120VC).
- Fine-tip tweezers dedicated to optical components (Thorlabs, Newton, NJ, USA, Cat. no.: TZ1).
- Thin, clean, lidded storage box (MUJI, Tokyo, Japan, Cat. no.: 4548076749066).
- Rotary tool fitted with a diamond-coated abrasive disk—Dremel 3000 (Dremel, IL, USA, Cat. no.: 3000-1/25).
- Safety glasses (Gigant, Moscow, Russia, Cat. no.: 34930561).
- Particulate respirator suitable for fine ceramic dust (3M, St. Paul, MN, USA, Cat. no.: 70070709012).
- Local dust-extraction system (3M, St. Paul, MN, USA, Cat. no.: 64193).
- LC-compatible polishing puck (Thorlabs, Newton, NJ, Cat. no.: D50-LC).
- Heat gun-LUKEY 852D+ (LUKEY, Guangzhou, China, Cat. no.: 814522).
- Manual ruby fiber cleaver or equivalent scribing tool (Thorlabs, Newton, NJ, USA, Cat. no.: S90R).

## 3. Procedure

### 3.1. Fabrication of Fiber-Optic Implants (30 min/10 pcs, 60 min, 10 min/10 pcs)

The reconnectable fiber-optic interface consists of two components: (1) a short implantable fiber segment mounted in an LC/PC-compatible ceramic ferrule and (2) an approximately 1.5 m long connecting fiber used to couple the implant to the optical setup. Both components are fabricated from step-index multimode optical fiber with core and cladding diameters of 105 and 125 µm, respectively (FG105UCA, Thorlabs, Newton, NJ, USA). During implantation, only the short ferrule-mounted segment remains chronically attached to the animal, whereas the long connecting fiber is attached through a ceramic LC mating sleeve only during optical measurements. A separate, factory-polished commercial LC/PC ferrule assembly is used during fabrication of the short implantable component solely as a temporary axial stop. The optical-fiber type in this temporary assembly is not critical. For example, a commercial patch cable based on FG050LGA fiber may be used. Hereafter, the ferrule end that is coupled to the long connecting fiber is referred to as the connector side, and the opposite end, from which the implantable fiber protrudes, is referred to as the tissue-facing side. Commercial implantable fiber-optic cannulas are available from manufacturers such as Doric Lenses and RWD Life Science. Selection should consider compatibility with the long connecting fiber and optical setup, particularly the fiber core diameter, numerical aperture, and ferrule–sleeve connection, together with the required protruding fiber length. The in-house fabrication procedure described below provides an alternative based on standard components, intended to reduce consumable costs and allow implant lengths to be tailored to the experiment.

#### 3.1.1. Fabrication and Assembly of the Implantable Fiber Segment (30 min/10 pcs)

**Figure 2:**
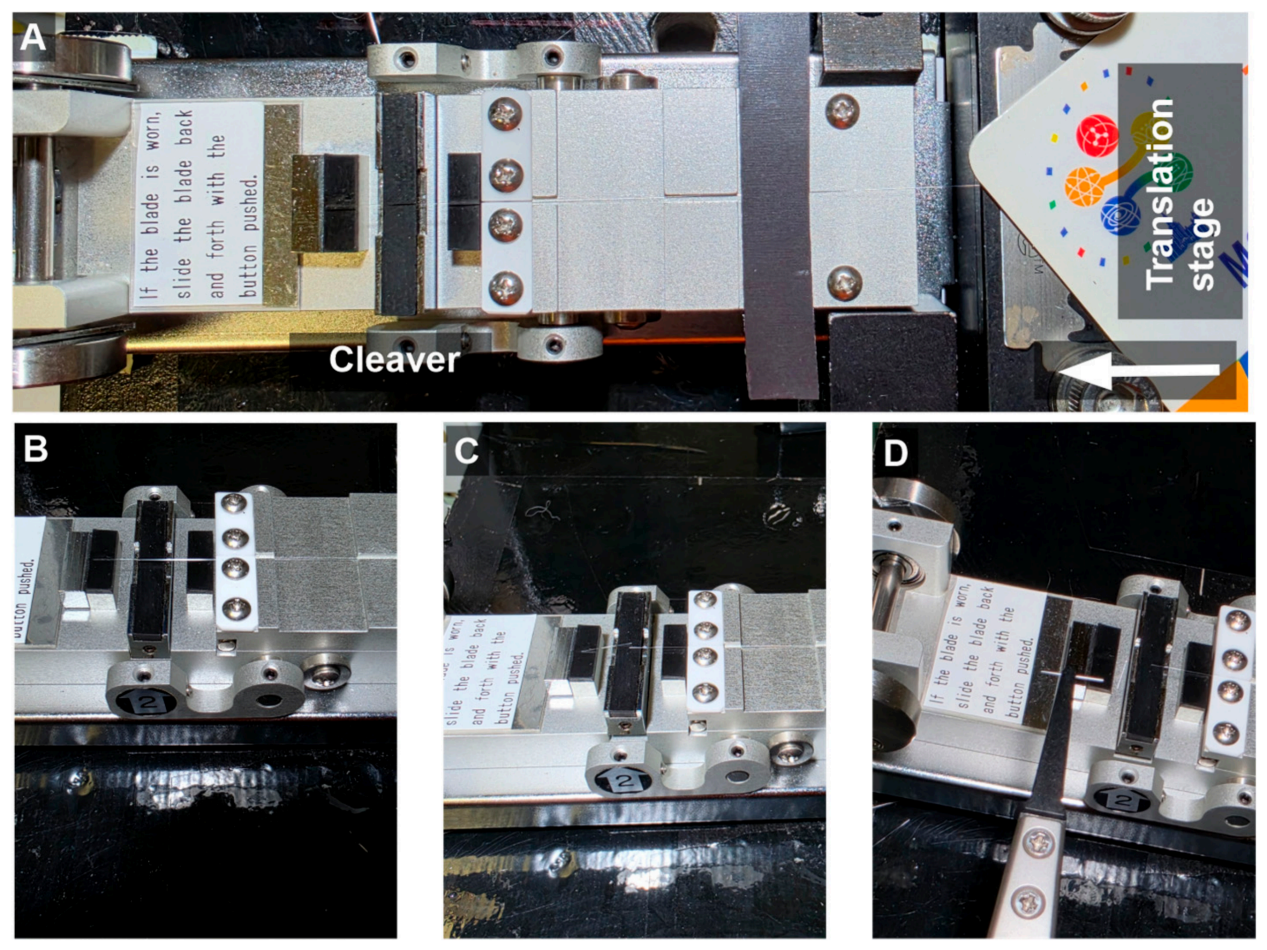
Preparation of short implantable fiber segments using the custom cleavage setup. (**A**) Top view of the custom-built cleavage frame combining the fiber cleaver and motorized translation stage. The fiber is fixed with a magnetic clamp. (**B**) Stripped optical fiber positioned in the cleavage setup before cutting. (**C**) Pre-cut short bare-fiber segment after the second cleavage. (**D**) Retrieval of the pre-cut segment with plastic fine-tip tweezers.

1. Prepare a clean fiber-processing workspace equipped with a stereomicroscope. Arrange the FG105UCA optical fiber, fiber stripper, fiber cleaver, computer-controlled translation stage, ceramic ferrules, ceramic LC (Lucent-connector-type) mating sleeves, temporary factory-polished ferrule assembly, NOA61 optical adhesive, UV-curing source, and clean fine-tip tweezers.
2. Cut a section of FG105UCA fiber long enough to fabricate the planned batch of implants, allowing additional length for handling and any necessary repeat cleaves. Remove the acrylate coating from the portion to be used to produce implantable segments.
3. Wipe the stripped fiber with isopropyl alcohol.
4. Place the stripped fiber in the custom-built fiber-cleavage frame equipped with a fiber cleaver (XL411, Thorlabs, Newton, NJ, USA) and a computer-controlled motorized translation stage (MT1/M-Z8, Thorlabs, Newton, NJ, USA) (Figure 2).
5. Cleave the stripped fiber to produce the first flat endface. This endface will subsequently form the connector-side end of the implantable segment.
6. Set the distance between the first endface and the second cleavage plane to obtain the required total segment length. The total segment length should include both the portion located within the ferrule and the required tissue-facing protruding length.
7. Perform the second cleavage. The custom-built cleavage frame allows the segment length to be set with an accuracy of approximately 20 µm (Figure 2C).

**Note:** If the custom-built cleavage system is unavailable, short fiber segments can be prepared using a manual fiber cleaver (e.g., S90R, Thorlabs). The principal advantage of the custom-built system is accurate and reproducible control of segment length. For manually cleaved segments, verify the protruding length of each completed implant under a stereomicroscope and confirm that it meets the experimental requirements.

8. Carefully collect the resulting short bare-fiber segment using clean fine-tip tweezers (Figure 2D). Hold the segment only by its lateral surface and avoid contact with either cleaved endface.

**Note**: Do not touch the cleaved endface with tweezers or other instruments. Contamination introduced at this stage cannot be removed after the short segment has been fixed inside the ferrule.

**Note:** Representative total lengths of the pre-cut implantable segments are 7.3 mm for cortical implantation and 11.7 mm for striatal implantation in the brain of 2-month-old rats, corresponding to tissue-facing protruding lengths of 1.8 mm and 5.2 mm, respectively. Other target-specific lengths can be produced by changing the total segment length before the second cleavage.

**Figure 3:**
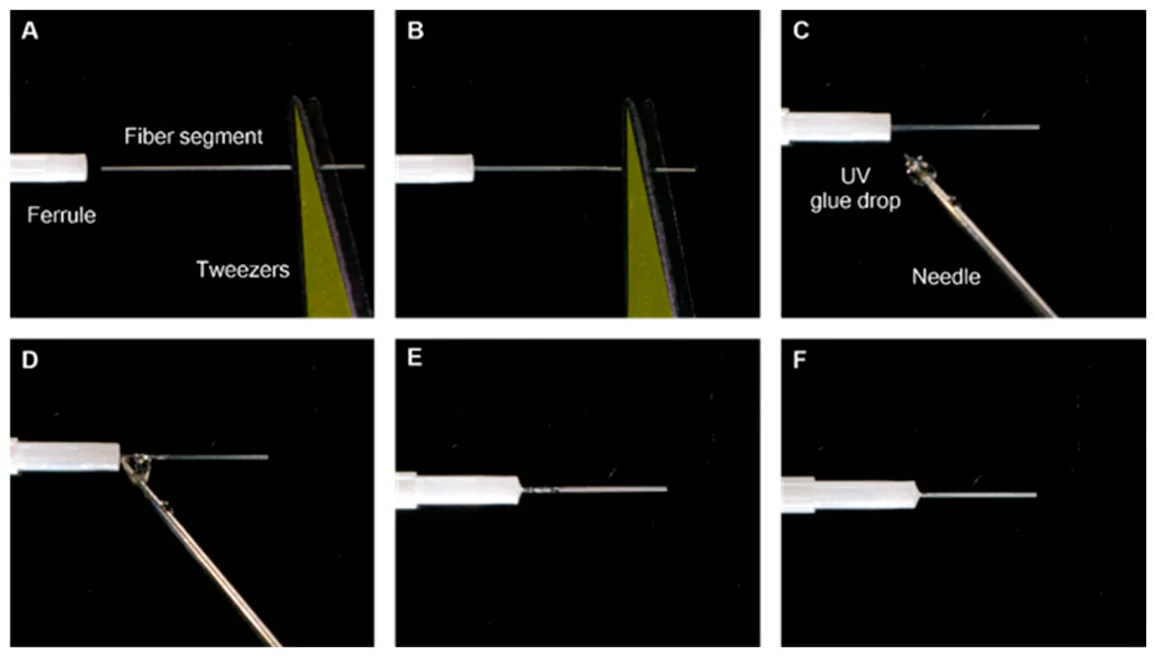
Assembly of the short implantable fiber segment in the ceramic ferrule. (**A**) Positioning of the pre-cut fiber segment with tweezers before insertion into the implant ferrule. (**B**) Partial insertion of the pre-cut fiber segment into the implant ferrule through the tissue-facing side. (**C**) Adhesive applicator (needle) carrying a small droplet of NOA61. (**D**) Application of NOA61 to the annular gap between the fiber and the ferrule. (**E**) Final advancement of the fiber segment until contact with the temporary axial stop. (**F**) Completed implant after UV-curing of the adhesive.

**Figure 4:**
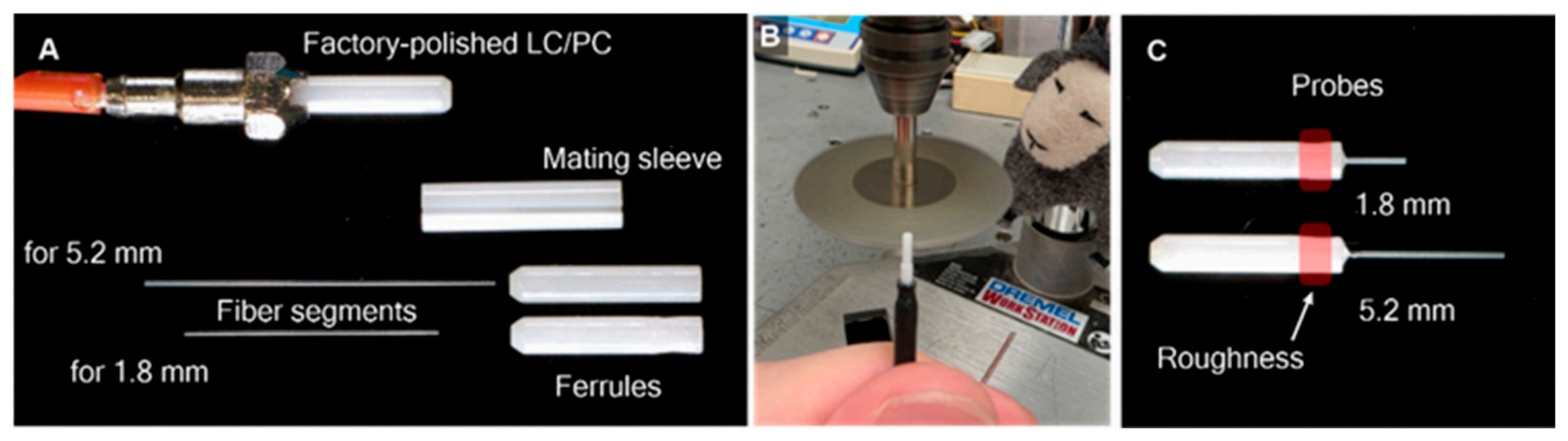
Additional fabrication steps and final appearance of the implantable probes. (**A**) Components used to fabricate the implantable probe, including the temporary axial-stop assembly, ceramic mating sleeve, implant ferrule, and pre-cut fiber segment. (**B**) Local roughening of the lateral ceramic surface near the tissue-facing end of the ferrule using a diamond-coated abrasive disk. (**C**) Completed implantable probes. The roughened region used to improve mechanical retention within the dental-cement headcap is indicated.

9. Insert the factory-polished LC/PC ferrule assembly used as the temporary axial stop into one side of a clean ceramic LC mating sleeve (Figure 3A, Figure 4A).
10. Insert an empty implant ferrule into the opposite side of the mating sleeve, with its connector side facing the factory-polished assembly stop. Ensure that both ferrules are fully seated in the sleeve.
11. Insert the pre-cut FG105UCA fiber segment through the tissue-facing side of the implant ferrule, but do not advance it fully. Stop before the connector-side endface of the fiber reaches the temporary factory-polished axial stop, leaving sufficient fiber exposed for controlled handling (Figure 3B).
12. Using a fine needle or a short optical-fiber offcut as an adhesive applicator, apply a small droplet of UV-curable optical adhesive (NOA61) at the annular gap between the partially inserted fiber and the tissue-facing opening of the ceramic ferrule (Figure 3C,D).
13. Carefully advance the fiber segment until its connector-side endface comes into direct contact with the polished endface of the temporary axial stop. As the fiber is advanced, allow the adhesive to wet the interface between the fiber and the ferrule channel (Figure 3E).

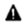

**CRITICAL STEP.** Use only the minimum amount of adhesive required to fix the fiber. Excess adhesive may be displaced toward the connector-side endface as the fiber is advanced, potentially contaminating the optical contact or bonding the implant ferrule to the mating sleeve or temporary axial stop.

14. Maintain the fiber in contact with the temporary axial stop and cure the adhesive using a handheld UV source according to the adhesive manufacturer’s instructions until complete polymerization (Figure 3F).

15. Hold the implant ferrule and carefully remove it from the ceramic mating sleeve. Remove the temporary factory-polished axial stop separately.
16. For each production batch, inspect the first completed implant under the microscope and verify the tissue-facing protruding fiber length. If the measured length differs from the target value, adjust the total segment-length setting on the cleavage frame before assembling the remaining implants. Once the cleavage and assembly settings have been verified, fabricate the remaining implants in the batch using the same settings without routine microscopic measurement of every individual implant.

**Note:** Neither endface of the short implantable fiber segment is mechanically polished. Both endfaces are formed by precision cleavage before the segment is inserted into the ferrule.

17. Using a rotary tool fitted with a diamond-coated abrasive disk, lightly roughen a small region of the external lateral surface adjacent to the tissue-facing end of the ceramic ferrule (Figure 4B,C). This treatment improves mechanical retention of the ferrule in the dental-cement headcap during subsequent implantation.

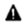

**CRITICAL STEP.** Wear safety glasses and a properly fitted particulate respirator, and perform this operation under local dust extraction to minimize exposure to airborne ceramic dust.

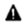

**CRITICAL STEP.** Roughen only the external lateral ceramic surface.

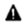

**CRITICAL STEP.** Do not contact the protruding optical fiber, either ferrule endface, or the central channel, and avoid excessive pressure that could crack the ferrule or break the fiber.

#### 3.1.2. Fabrication of the Long Connecting Fiber (60 min)

1. Cut an approximately 1.5 m -long segment of FG105UCA fiber.
2. Feed the fiber through a protective furcation tube, leaving sufficient free fiber length at both ends for stripping, cleavage, and connector assembly.
3. Remove the acrylate coating from the animal-facing end over a length sufficient for insertion into an LC/PC-compatible ceramic ferrule. Cleave the stripped end to obtain a clean endface.
4. Apply a small amount of NOA61 to the stripped animal-facing end of the fiber and insert it into the ceramic ferrule. Advance the fiber until it protrudes slightly beyond the ferrule endface.
5. Cure the adhesive under UV illumination according to the manufacturer’s instructions until complete polymerization.
6. Remove the excess fiber protruding from the ferrule using a manual ruby fiber cleaver or an equivalent fiber-scribing tool.
7. Place the ferrule in an LC-compatible polishing puck and manually polish the endface using a sequence of progressively finer polishing films. Continue polishing until the fiber is flush with the ferrule surface and the endface is free of visible adhesive residue, chipping, and major scratches.

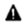

**CRITICAL STEP.** Keep the ferrule perpendicular to the polishing surface. Uneven pressure or tilted polishing can produce an oblique endface and reduce optical coupling to the implantable segment.

8. Inspect the polished ferrule endface under the microscope. Repeat the final polishing step if visible defects or polishing residue remain.
9. Pass an approximately 5-cm-long piece of adhesive heat-shrink tubing over the junction between the ceramic ferrule and the furcation tube. Shrink the tubing using a heat gun to provide mechanical strain relief.
10. Prepare the setup-facing end of the long fiber according to the coupling geometry of the optical system. For free-space coupling, leave this end bare and freshly cleaved. When several connecting fibers must be positioned in a common focal plane, align and secure their setup-facing ends using the laboratory-specific multichannel fiber-holder arrangement.
11. Protect the polished ferrule end from dust and mechanical damage until optical testing and use.

#### 3.1.3. Optical Quality Control and Storage (10 min/10 pcs)

1. Couple 473-nm light into the setup-facing end of the completed long connecting fiber.

**Note:** In our work, we used biosensors based on yellow fluorescent protein. Light at any wavelength of interest can be used.

2. Position the S120VC photodiode power sensor connected to the PM100D optical power meter (Thorlabs, Newton, NJ, USA) at the output of the polished ferrule of the long connecting fiber.
3. Record the reference optical power, *P*_long_. Place the detector sufficiently close to the ferrule to collect the full divergent output cone.
4. Insert the polished ferrule of the long connecting fiber into one side of a clean ceramic LC mating sleeve.
5. Insert the completed implant ferrule into the opposite side of the mating sleeve. Ensure that both ferrules are fully seated.
6. Without changing the 473-nm source power, the coupling conditions at the setup-facing end, or the power-meter settings, measure the optical power at the tissue-facing tip of the implantable fiber, *P*_assembled_.
7. Calculate the transmission introduced by the ferrule–sleeve interface and implantable segment as *T* = *P*_assembled_/*P*_long_ * 100%.
8. Use only implants that pass the initial post-fabrication transmission test *T* > 85% for implantation and subsequent optical measurements.

**Note:** The *T* > 85% threshold applies to initial post-fabrication quality control and should not be interpreted as the typical transmission during photometry. For subsequent experimental measurements, use an index-matching gel (G608N3, Thorlabs) between the mating fiber endfaces when connecting the long fiber to the implanted probe.

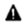

**CRITICAL STEP.** A low transmission value may result from contamination of the fiber endfaces. If *T* ≤ 85%, inspect all accessible fiber endfaces, particularly the mating endfaces of the long connecting fiber and the implant, for contamination. Clean the endfaces with a lint-free optical wipe, reconnect the components, and repeat the transmission measurement without changing the source-to-fiber coupling conditions or the power-meter settings. Reject the implant if *T* remains ≤ 85% after cleaning and remeasurement.

9. Place the implant horizontally in a thin, clean, lidded storage box. Secure only the lateral surface of the ceramic ferrule to a strip of double-sided adhesive tape.
10. Ensure that the protruding fiber and its tissue-facing endface do not contact the adhesive tape, the bottom of the box, or adjacent implants.
11. Insert and remove implants using clean fine-tip tweezers, touching only the ceramic ferrule. Keep the storage box closed between procedures.

### 3.2. Surgical Manipulations: Injection of an AAV Suspension Carrying the Biosensor Gene and Optical-Fiber Implantation (60 min, 20 min/Injection Site, 5 min/Ferrule, 10 min, 30 min)

**Figure 5:**
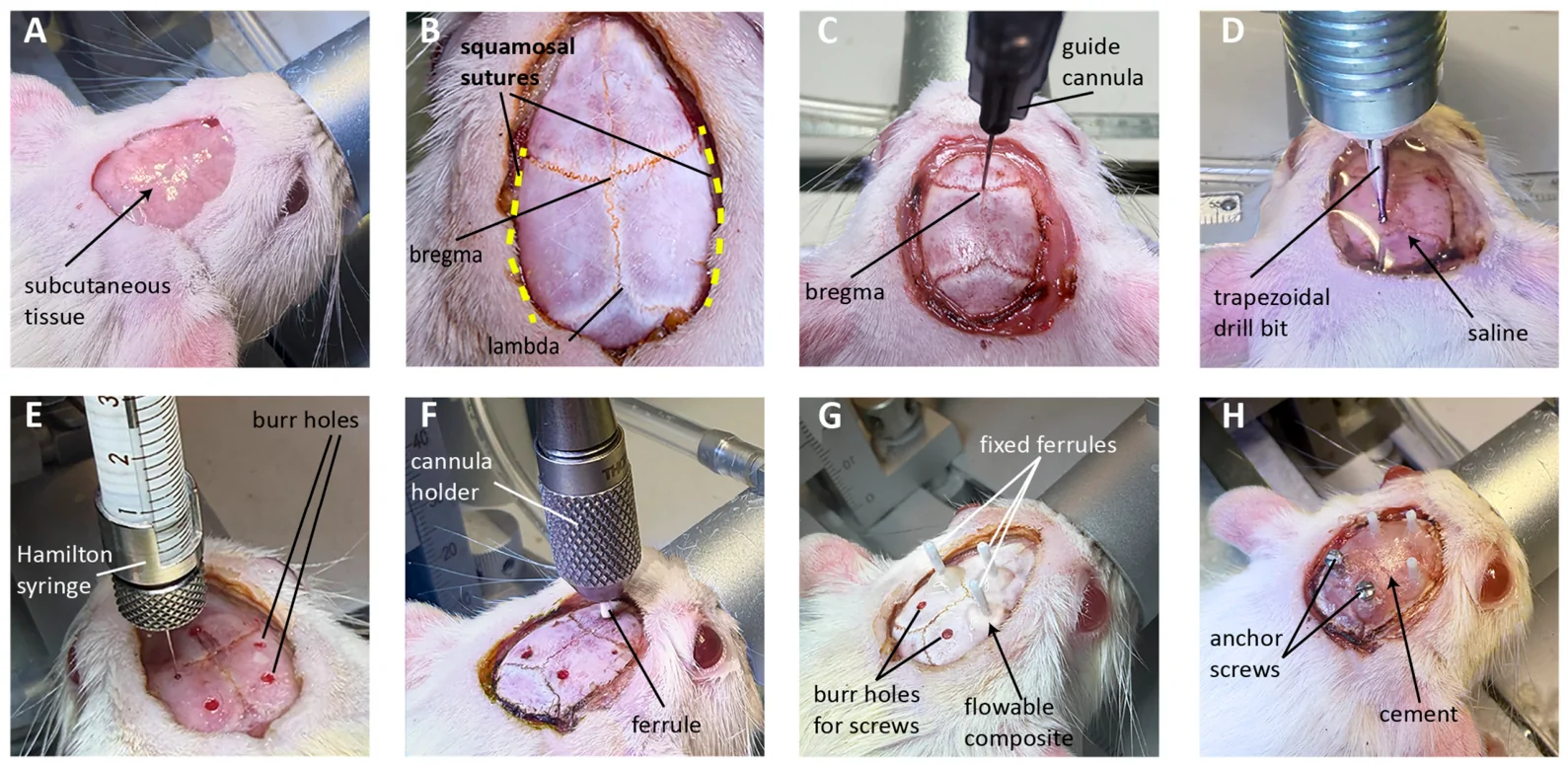
Main steps of the surgical procedures, including injection of viral particles into the brain tissue and implantation of optical fibers. (**A**) Surgical skin excision and preparation of surgical field. (**B**) Tissue removal and preparation of the skull surface. (**C**) Location of the bregma and marking of the injection sites according to the selected coordinates. (**D**) Drilling according to coordinates. (**E**) Injection of viral particles into the brain using a Hamilton syringe. (**F**) Implantation of optical fibers. (**G**) Fixation of the ceramic ferrules to the skull with a UV-curing composite. (**H**) Overall view of the headcap construction after placement of screws and curing of the composite securing the ferrules.

Surgical procedures used to prepare animals for photometry include preparation of the surgical field, injection of a suspension of viral particles carrying the gene encoding the selected biosensor into the tissue at predefined brain coordinates, and implantation of optical fibers with the ceramic ferrules secured to the skull using dental cement. Screws are used to secure the assembly to the skull. The fixed fibers remain in place for several months, and the animals tolerate this procedure well, with the implants having no apparent adverse effects on their normal functioning. All procedures must be approved by the Institutional Animal Care and Use Committee (IACUC). This protocol is designed for rats, but it can be adapted for mice. The procedure requires the workspace to be equipped with a stereotaxic frame integrated with an inhalation anesthesia system, as well as a binocular operating microscope mounted on a stand and oriented toward the surgical field. The operative area is provided with strong illumination to ensure optimal visualization during all procedures. The entire procedure is performed under aseptic conditions. The sequential steps of the procedure are presented in Figure 5.

#### 3.2.1. Surgical Field Preparation (60 min)

##### Workspace and Equipment Preparation

1. Sterilize the surgical tools using an autoclave.
2. Clean the workspace with 70% ethanol and cover the surgical area with a sterile sheet.
3. Fill the vaporizer reservoir with isoflurane.
4. Lay out the minimal instrument set: a scalpel, curved tweezers, scissors, a cement spatula, a cement-mixing well, two insulin syringes, a fine trapezoidal drill bit, a wide drill bit, and the Hamilton syringe. Arrange all required reagents and consumables.
5. Store the AAV aliquots at +4 °C and remove them only immediately before loading the Hamilton syringe.
6. Remove the metal plunger from the Hamilton syringe. Attach a 27G needle to a 1 mL syringe filled with saline or PBS. Place the needle on the top part of the Hamilton syringe. Then flush the Hamilton syringe with the solution to make sure it is sealed and to remove all air bubbles. Insert the plunger back.
7. Prepare a 1% aqueous sodium hypochlorite solution in a container for the disposal of virus-contaminated plasticware.

##### Animal Anesthesia

1. Weigh the animal to determine the appropriate anesthesia parameters.
2. To induce anesthesia, place the animal in the inhalation chamber and begin isoflurane administration to 4–5% with an airflow of approximately 1 L/min. Monitor the animal’s breathing until it becomes slow and deep.
3. Once the animal is deeply anesthetized, stop the gas flow to the induction chamber. Transfer the animal to the stereotaxic apparatus. Gently open the jaw using tweezers and position it onto the bite bar so the upper incisors engage with the bar opening. Connect the inhalation mask, maintaining isoflurane at up to 2.5% (the airflow rate is adjusted based on the animal’s body weight, 350 mL/min for a 300 g rat).

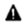

**CRITICAL STEP.** Before proceeding, confirm that a surgical plane of anesthesia has been reached by verifying the absence of the hindlimb withdrawal reflex. Continuously monitor the depth of anesthesia throughout the surgery. The respiratory rate should remain between 60 and 120 breaths/min; a rate below 60 breath/min indicates a critical situation requiring immediate attention.

4. Fix the head using the rat ear bars (or mouse ear bars in the case of procedures with mice). Ensure that the ear bars protrude equally from their housings on both sides (verify using the calibration scale) and are positioned at the same height. Fixation is adequate when gentle pressure applied with tweezers does not cause the skull to move.
5. Insert a rectal thermometer probe lubricated with petroleum jelly.
6. Apply ophthalmic ointment to the eyes to prevent them from drying out during the surgery.
7. Administer ketoprofen/flexoprofen subcutaneously at a dose of 5 mg/kg, diluted in saline (e.g., by injection into the scruff).

##### Skull Exposure

1. Shave the head of the rat from the eyes to the ears and disinfect the skin with povidone–iodine/alcohol.
2. Inject 300–500 µL of an anesthetic (e.g., 0.5% bupivacaine) subcutaneously at the incision site locally. Wait for 2–3 min to allow the anesthetic to spread through the tissue.
3. Make a single midline incision extending from between the eyes to between the ears. Excise a small, round section of skin to expose the skull (Figure 5A).
4. Remove the subcutaneous tissue and mechanically scrape all soft tissue from the bone using a bone scraper and cotton-tipped applicators (this step is not necessary for mice). The surgical field should not extend beyond the lateral borders of the parietal bones (squamosal suture, Figure 5B). In rats, it should extend approximately 4–5 mm rostral to bregma anteriorly and 3–4 mm caudal to lambda posteriorly. If necessary, small areas of the skin may be removed laterally.

**Note:** At this stage of the procedure on the mouse, it is recommended to make shallow crisscross incisions on the skull to roughen the surface, ensuring a strong bond with the headcap.

5. Apply 3% hydrogen peroxide to the skull surface to remove any remaining soft tissue using a scalpel and cotton-tipped applicators.
6. Apply a hemostatic agent to the surrounding tissue using a cotton-tipped applicator to control bleeding.
7. Degrease the skull surface with 70% ethanol.
8.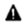 **CRITICAL STEP.** Coat the skull and wound margins with a thin layer of tissue adhesive (e.g., 3M™ Vetbond™ Tissue Adhesive) and allow it to dry. The next step can be started before the adhesive has fully dried (Figure 5B).

##### Skull Leveling and Craniotomy

1. Mount a guide cannula (e.g., an empty insulin syringe) onto the stereotaxic holder.
2. Locate bregma (the intersection of the coronal, sagittal, and frontal (metopic) sutures), place the guide cannula over this location, lightly touching the skull, and set to zero (or record) all coordinates (ML = 0, AP = 0, DV = 0). Bregma may be marked with a fine-tip marker. If the sutures are irregular, identify bregma as the midpoint of the gap between them. If the sagittal suture is crooked, follow the imagined line connecting the segments (Figure 5C). The acceptable error for all stereotaxic coordinates is ≤ 0.1 mm.
3. Level the rostro–caudal axis. Confirm that the needle follows the midline precisely as it moves from the bregma toward the lambda and that the bregma and lambda are at the same height (DV = 0). If any deviation is observed, reposition the animal and repeat this step. Adjust the nose height using the mask. If the lambda is displaced to the left or right of the bregma, reposition the ear bars accordingly.
4. Level the left–right axis at ML ± 0.9 and AP ± 4. If a difference in height is observed between these points, adjust the screws controlling the ear-bar height accordingly. Using a marker (acrylic or alcohol-based), mark the planned injection sites on the skull surface. Representative coordinates used in this protocol are provided in Table 1.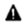 **CRITICAL STEP.** Before drilling, place a drop of saline over the site (Figure 5D). Start the drill without touching the skull, then gently bring it into contact with the bone within the saline droplet, resting the hand on the ear bar to keep it steady. To avoid damaging the dura, leave a thin layer of bone intact and carefully remove it with fine tweezers or complete the drilling at low speed. See Troubleshooting, Appendix A (Table A1).
6. Drill a burr hole at each marked site using a 0.5 mm trapezoidal drill bit.
7. Refill each hole with a drop of saline and ensure all holes remain covered with saline until the moment of injection.
8. In the posterior part of the parietal bone, approximately 2–3 mm lateral to the sagittal suture, drill a wider hole for the anchor screw and cover it with a small drop of saline. This step is optional for mice, as the headcap holds sufficiently firm after the soft tissue is completely removed, the skull surface is roughened, and the bone is treated with hydrogen peroxide.

#### 3.2.2. Stereotaxic Injection of Viral Particles (20 min/Injection Site)

1. Mount the flushed Hamilton syringe (paragraph “*Workspace and equipment preparation*”, point 6) in the stereotaxic holder and gently tighten the retaining screw. Set the pump parameters: the syringe model, target injection volume (1 μL) and pump flow rate. The pump flow rate should be selected according to the injection coordinate and the recommended values provided in Table 1. For the injection volumes used in this protocol, a flow rate of 100–200 nL/min is recommended as a practical range, consistent with rates commonly reported for AAV injections into the rodent brain [29,30,31].
2. Remove the required amount of viral suspension AAV from the refrigerator. Using a pipette, place a droplet of viral suspension onto a clean piece of Parafilm, calculated as 1 μL per injection site. Promptly position the Hamilton needle tip in the droplet and draw the suspension into the syringe. Once loading is complete, return the remaining viral suspension to the refrigerator. Dispose of all disposable plastic items (tips, test tubes, Parafilm) that have come into contact with the AAV suspension in a container containing 1% sodium hypochlorite.

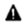

**CRITICAL STEP.** Based on our experience, the optimal titer range of the virus is 1 × 10^11^–1 × 10^13^.

3. At the injection site, position the needle tip on the skull surface and set the DV coordinate to zero. Throughout this protocol, DV coordinates for injection sites are referred to the skull surface. Then slowly insert the needle into the brain to the target DV coordinate at a rate of approximately 2 mm/min. Move the syringe an additional 0.1 mm and wait for 1 min.
4. Raise the needle again by 0.1 mm and begin the injection using the preset pump settings (Figure 5E).
5.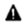 **CRITICAL STEP.** After completing the injection, wait an additional 5 min before withdrawing the needle. Then withdraw the needle slowly to avoid drawing the injected bolus back up the needle track.
6. Repeat steps 1–5 for each injection site.
7. Remove the Hamilton syringe from the holder and flush it sequentially with distilled water, 70% ethanol, and acetone. Allow the syringe and plunger to air-dry on a clean wipe for 10–15 min before returning to storage.

#### 3.2.3. Optical-Fiber Implantation (5 min/Ferrule)

1. Disinfect the ferrules with ethanol and allow them to air-dry.
2. Dry the skull surface around each burr hole before implantation.
3. Mount the ferrule in the cannula holder (Thorlabs) and zero the coordinates at the fiber tip.

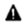

**CRITICAL STEP.** Avoid pressing the tip directly against the skull.

4. At the injection coordinates, set the DV coordinate to zero at the fiber tip on the skull surface, then slowly insert the ferrule to the target depth, positioning the implantable fiber tip 0.3 mm dorsal to the injection DV coordinate. In the protocol we use, the implantation depth is 1.8 mm for the cortex and 5.2 mm for the striatum. If bleeding occurs, pause advancing the fiber into the tissue, wait for the bleeding to stop, clear blood from the skull surface, then complete the implantation (Figure 5F).
5. Secure the ferrule to the skull surface using a light-curing flowable composite, ensuring that the composite is applied continuously around the entire circumference of the ferrule base. Ensure that the composite does not extend beyond the cleared skull margin or contact with the surrounding soft tissue. Cure with UV light for 20 s.
6. Release the ferrule from the holder and only then raise the holder.
7. Repeat steps 2–6 for all ferrules (Figure 5G).

#### 3.2.4. Headcap Construction (10 min)

1. Place the anchor screw perpendicularly to the skull surface. Hold the screw with tweezers and use a screwdriver to turn it 1–1.5 turns until it is firmly sited in the bone.

**Note:** Optionally, anchors can be implanted before the ferrules to avoid accidental displacement of the ferrules by the screwdriver.

2. Mix the headcap cement by gradually adding the powder 350–500 μg to the liquid component of about 350 μL until the mixture begins to thicken. Stir for 30–60 s until the desired consistency is achieved.
3. Cover the entire skull surface with cement, taking care not to flood the surrounding soft tissue. Completely embed the screw and leave 3–4 mm of each ferrule exposed at the top. If cement reaches the upper part of a ferrule, carefully remove it with a scalpel before it sets.

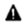

**CRITICAL STEP.** If the cement extends onto soft tissue, carefully remove it and ensure that a clear gap remains between the cement cap and the surrounding tissue (Figure 5H).

#### 3.2.5. Recovery, Post-Operative Care (30 min)

**Figure 6:**
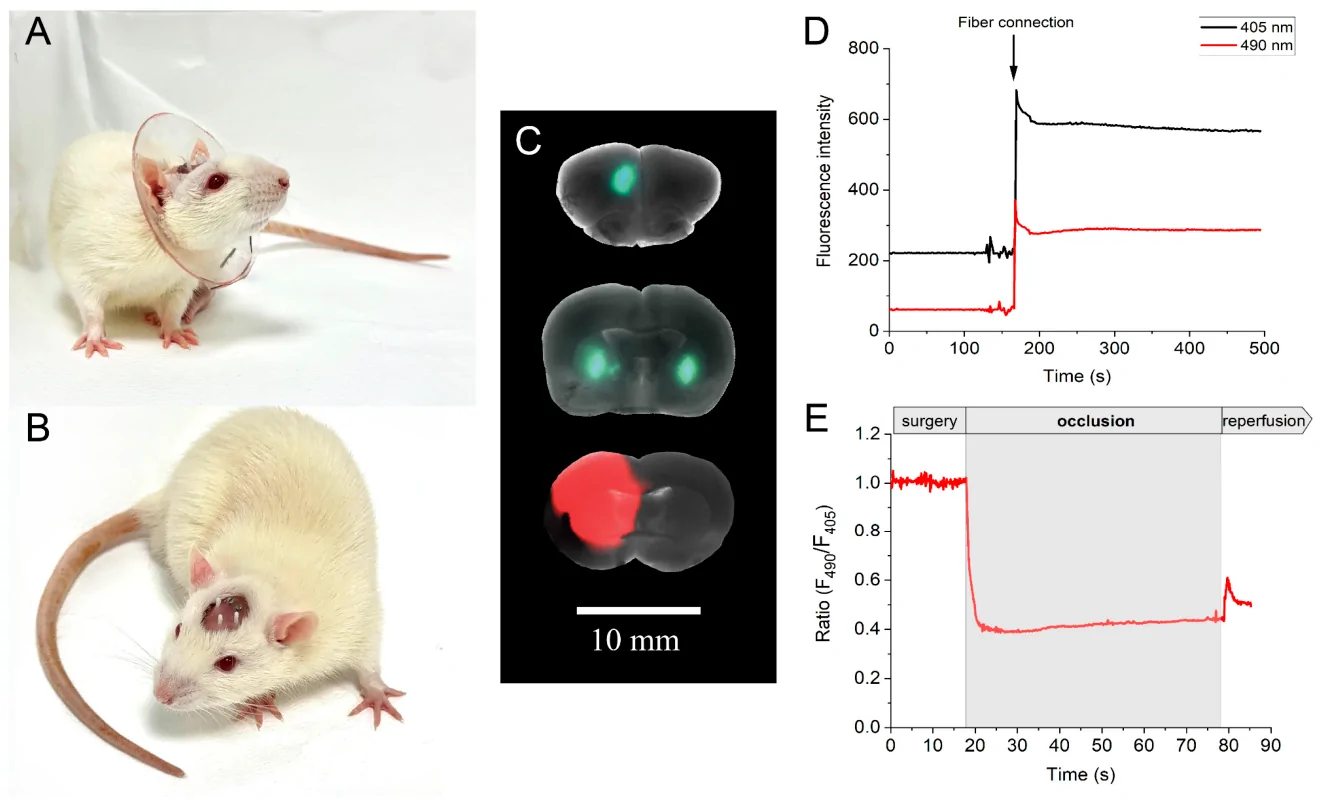
Results of the surgical procedure. (**A**) An operated-on rat wearing the protective collar during the first few days after surgery. (**B**) A healthy animal with the three-fiber chronic implant one month after surgery. Ceramic ferrules with optical fibers are visible on the skull surface secured with dental cement. (**C**) Photographs of brain slices from a rat showing SypHer3s gene expression in the cortex (AP 3.2 mm, ML 1.0 mm, DV −2.1 mm, upper slice) and in the striatum of both hemispheres (AP −0.9 mm, ML ±4.0 mm, DV −5.5 mm, middle slice). The fluorescent areas correspond to regions expressing the biosensor gene. The bottom slice shows the fluorescence of the red protein mRuby2 as an illustrative example demonstrating that a more extensive brain region expressing the fluorescent protein can be achieved by injecting the increased volume of the viral suspension into multiple sites along the dorsoventral axis (DV −5.5, −4.5, −3.5, −2.5, −1.5 mm). (**D**) Recordings of fluorescence signals in the channels with excitation at 405 nm and 490 nm before and after connection of the fiber-photometry setup to the implanted probes in the brain tissue of a rat expressing the SypHer3s biosensor gene one month after surgery. The recordings clearly demonstrate that the SypHer3s signal is substantially higher than the background level. (**E**) Dynamics of the SypHer3s signal, calculated as fluorescence intensities ratio F490/F405, in rat brain tissue in vivo during ischemia/reperfusion in the MCAO model.

1. To protect the implant and wound from scratching, trim the animal’s claws and fit a protective collar. The collar is made from a sheet of clear PVC tablecloth (soft, transparent PVC film). Cut out a ring with an outer diameter of approximately 7.5 cm and an inner diameter of approximately 3 cm. Make a single radial slit from the inner opening to the outer edge so it can be placed around the neck. Adjust the collar so that it cannot slip over the skull and be removed by the rat, while ensuring that it remains loose enough to avoid compressing the neck. Secure the collar with a stapler. An example of an animal wearing the protective collar is shown in Figure 6A. Keep the collar on the animal for the first 3–7 days to prevent injury, after which it can be removed.

**Note:** Rats require a protective collar to prevent them from pulling out the headcap and implanted fibers during grooming or forepaw manipulation, as this is a common cause of implant loss. This is generally unnecessary in mice.

2. Remove the ear bars and transfer the rat to a clean cage with bedding. An infrared lamp may be used to provide warmth, but it must not be directed at the animal (overheating risk).

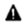

**CRITICAL STEP.** Do not leave the rat under the lamp for extended periods.

3. Provide ketoprofen in the drinking water at a dose of 5 mg/kg/day for up to 3 days.
4. Place moistened chow on the cage floor.
5. After one week, the animal can be returned to standard housing conditions. For most fluorescent biosensors, a detectable level of signals in the brain tissue is observed 2–3 weeks after surgery.

## 4. Expected Results

Here, we present a detailed and reproducible protocol for multi-site AAV delivery into the Norway rat brain, followed by chronic implantation of optical fibers securely fixed at three distinct target brain regions. We also provide a comprehensive description of the custom fabrication of implantable optical fibers, covering the entire workflow from raw optic fiber to a functional implant.

The optical interface has a modular two-part design comprising a short implantable fiber segment mounted in an LC/PC-compatible ceramic ferrule and a reconnectable longconnecting fiber approximately 1.5 m in length. The long connecting fiber is fabricated separately, terminated with an LC/PC-compatible ceramic ferrule on the animal-facing side, and coupled to the implant through a ceramic LC mating sleeve during optical measurements. Both sections are based on FG105UCA step-index multimode optical fiber with core and cladding diameters of 105 and 125 µm, respectively, and are coupled through a ceramic LC mating sleeve. The patch cable is not implanted and is attached only during implant assembly, optical quality control, and subsequent measurement sessions. The proximal endface of the implantable fiber segment is used directly after cleavage and does not require mechanical polishing. The protruding fiber length is selected according to the depth of the anatomical target. Representative lengths used in this protocol are 1.8 mm for cortical implantation and 5.2 mm for striatal implantation. Following the fabrication workflow described in Section 3.1, the optical probes were produced reproducibly in batches. Each manufactured implant was subjected to optical quality control before the surgical procedure, and only probes with a transmission of >85% at the ferrule–sleeve interface were accepted for the experiment (60–70% of probes met this criterion). The resulting optical probes were compatible both with commercial fiber-photometry systems and a custom-built optical setup. The skull-mounted adapter features a universal design suitable for use in both rats and mice, while the length of the implanted fiber can be adjusted to the target depth in the brain.

Although stereotaxic delivery of AAV particles and chronic optical-fiber implantation have been extensively used in mice, analogous protocols for rats remain comparatively less standardized and are less frequently reported in dedicated methodological detail. Moreover, protocols and equipment may require species-specific adaptations to accommodate differences in animal size and strength; for example, reinforced optical fiber patch-cords have been developed specifically for use in rats [32]. Many studies, with the use of optogenetic and photometric technologies, have focused on single-site targeting rather than multi-site approaches. In our protocol, we describe rat-specific procedural details, a multi-target configuration, and an integrated multi-ferrule configuration in which a single headcap stabilizes all implants simultaneously.

The surgical procedures described here are well tolerated by the animals. General health status, body weight, and behavior remain within normal ranges, with no signs of infection or inflammation observed when the procedures are performed under aseptic conditions. In our experience, the use of a protective collar prevents damage to the implants and promotes better wound healing (Figure 6A). The implanted optical fibers and the composite headcap with ferrules remained mechanically stable throughout the chronic period (Figure 6B). No displacement or loss of the implants was observed. The construct remained intact, and the bone surface beneath the cement was not damaged.

In a series of our published studies [22,23,24,25,26], we have used this protocol to prepare rats for experiments in which fluorescent signals of biosensors were recorded in vivo during the development of ischemic stroke in a MCAO model. The ability to simultaneously record signals from multiple sites allows, for example, comparison of the dynamics of the same biochemical parameter in the tissues of the injured and healthy hemispheres. As an illustrative example for this protocol, we used a coordinate (AP −0.9; ML +4; DV −5.5) in the striatum that falls within the central ischemic core following middle cerebral artery occlusion in 3-month-old rats. The coordinate (AP +3.2; ML +1; DV −2.1) is located in the cortex and falls within the ischemic penumbra. Thus, this approach allows for monitoring of the dynamics of pathological processes with spatiotemporal resolution at the whole-brain scale.

In our work, we used a biosensor HyPer7 for hydrogen peroxide [3] or a pH-biosensor SypHer3s [27]. Two to three weeks after injection of viral particles carrying the HyPer7 or SypHer3s gene, a stable fluorescent signal can be detected at the specified brain coordinates, as confirmed by visualization in brain slices (Figure 6C). For these biosensors, the fluorescent signal can be detected in brain tissue for up to 12 months. In order to achieve stable and spatially extensive biosensor expression, we used AAV9 vectors for intraparenchymal delivery [33,34]. For stereotaxic injections, a suspension of viral particles with a titer ranging from 1 × 10^11^ to 1 × 10^13^ VG/ml is utilized. The volume of tissue expressing the biosensor gene can be further increased by distributing the whole volume of viral suspension across multiple injection sites along the dorsoventral axis. This approach also allows for the use of a larger total injection volume while minimizing the volume delivered at each individual site. In the example shown in Figure 6C (bottom slice showing the fluorescence of the red protein mRuby2 [35]), 0.3 µL was injected at each of five sites spaced 1 mm apart (DV −5.5 to −1.5 mm from the skull surface) for a total volume of 1.5 µL. The biosensors we used in our studies are based on a fluorescent core cpYFP characterized by two peaks in the fluorescence excitation spectrum. Thus, the measurements are performed in two channels, and the signal is calculated as the ratio of their fluorescence intensities. Figure 6D shows the dynamics of the SypHer3s fluorescence signal in the channels with excitation at 405 nm and at 490 nm before and after connecting the optical cables to the implanted probes in the tissue region expressing the biosensor. Figure 6E demonstrates the typical dynamics of the SypHer3s signal, calculated as F490/F405 ratio, in the central zone of brain tissue damage during ischemia/reperfusion, reflecting the development of severe intracellular acidosis.

Representative excitation powers used in our experiments were on the order of a few microwatts, measured at the tissue-facing tip of the implant with the excitation source operating continuously. Excitation power should be selected together with illumination duration, acquisition frequency, and total recording duration, taking into account the photostability of the chosen biosensor and the need to obtain sufficient fluorescence while limiting photobleaching.

The protocol presented here can readily be adapted for studies using any other disease models. For example, we used a similar approach to monitor biochemical events in rat brain tissue in a nitroglycerin-induced migraine model [36]. However, in most cases, the coordinates and strategy for fiber implantation should be optimized and validated for each specific application, as these parameters depend on the animal species, strain, and age, as well as on the brain regions from which the signal is to be recorded [37,38]. The implant fabrication protocol described here allows the fiber length to be adjusted, enabling signal recording from virtually any depth within the brain tissue. At the same time, the external adapter of the implant is universal and can be used in mice, further expanding the potential applications of this technology. Such approaches are compatible with a wide range of genetically encoded tools with fluorescent readouts and can be integrated with both commercially available and custom-made hardware configurations [39]. The multichannel design provides independent optical access to several brain regions and is compatible with simultaneous or sequential recording, depending on the optical configuration.

The quality of the outcome depends on several critical factors, including (1) accurate targeting of the coordinates and correct leveling of the skull; (2) thorough cleaning and adhesive treatment of the skull surface; (3) slow needle insertion; (4) a mandatory 5 min waiting period before needle withdrawal to prevent backflow of the suspension along the needle track; and (5) the stability of the composite and headcap, which is also essential. The genetic constructs and the quality of the viral particles carrying them should be tested before the main series of experiments. A number of limitations should also be taken into account. In some cases, implantation of probes may be constrained by the limited space on the animal’s skull. Recording from closely spaced coordinates in the brain can be challenging due to optical crosstalk, as well as the difficulty or inability to position the ferrules close enough to each other and connect the optical cables to them. A minimum distance of 3–4 mm should be maintained between ferrules. After each experiment, the expression of the genetic tool, the correct fiber localization, and the absence of surgically induced inflammatory reactions should be confirmed.

## Figures and Tables

**Table 1:** Representative stereotaxic coordinates for viral injection/optical-fiber implantation sites.

Injection Site	AP (mm)	ML (mm)	DV (mm) from the Skull Surface	Injection Bolus Volume, nL	Pump Flow Rate, nL/min
Left striatum (stroke lesion core)	−0.9	+4.0	−5.5	1000	200
Right striatum (contralateral hemisphere)	−0.9	−4.0	−5.5	1000	200
Frontal cortex (penumbra)	+3.2	+1.0	−2.1	1000	100
Dorsovental sequential injection (for widespread expression)	−0.9	±4.0	−5.5; −4.5; −3.5; −2.5; −1.5	300 each site	200

Coordinates should be verified and adjusted according to the specific experimental design. Pilot dye injections are recommended before beginning a full experimental cohort, as described for other stereotaxic models.

## Data Availability

The raw data and details supporting the conclusions of this article will be made available by the authors on request.

## Abbreviations

The following abbreviations are used in this manuscript:

AAV	Adeno-associated virus
AP	Anterior–Posterior
cpYFP	Circularly Permuted Yellow Fluorescent Protein
DV	Dorsal-Ventral
FRET	Fluorescence Resonance Energy Transfer
IACUC	Institutional Animal Care and Use Committee
LC	Lucent Connector (type)
MCAO	Middle Cerebral Artery Occlusion
ML	Medial–Lateral
NOA	Norland Optical Adhesive
PBS	Phosphate-Buffered Saline
PC	Physical Contact
PVC	Polyvinyl Chloride
UV	Ultraviolet
VG/ml	Vector Genomes per milliliter

## Appendix A

**Table A1:** Troubleshooting table.

Step	Problem	Possible Reason	Solution
Probe fabrication	Low transmission value (under 80%)	Optical-fiber damage or endface contamination	Inspect the optical fiber for damage and ensure that the fiber endfaces are not contaminated. If transmission remains low, reject the implant.
Craniotomy	Bone dust or debris in the burr hole	Drilling without saline irrigation	Drill holes in short pulses with periodic saline irrigation; wash away debris before injecting the viral suspension; cover the craniotomy site with a drop of saline while working on other areas.
Craniotomy	Bleeding after drilling a burr hole/during needle or fiber insertion	Damage to a meningeal blood vessel	Avoid craniotomy over the major dural venous sinuses; map surface vessels before inserting the needle or fiber; if your experimental design permits, shift the coordinates laterally by 50–100 µm, control bleeding with sterile saline irrigation, place a haemostatic sponge and wait until coagulation.
Viral suspension injection	Backflow of viral suspension along the needle track	Injection rate is too high; the needle was lifted too early	Reduce the rate of infusion; leave the needle in place for 5–10 min after the infusion, then withdraw slowly (e.g., 1–2 mm/min).
Viral suspension injection	Weak or absent sensor expression	Low viral titer	Verify the titer and use a serotype validated for the region and cell type.
Viral suspension injection	Weak or absent sensor expression	Serotype or promoter unsuited to the target cell type	Allow at least 3 weeks of expression before checking the level of fluorescence.
Viral suspension injection	Weak or absent sensor expression	Off-target coordinates	Refine the coordinates in a pilot group of animals using a dye or histological analysis before the main experiment.
Fiber implantation	Fiber bends or fails to enter the tissue	The skull was drilled through only partially; the fiber tip is pressing into the bone	Slightly widen the burr hole; lower the probe slowly and pause if you feel resistance; never force along the same track.
Headcap assembly	Dental cement or adhesive contamination of the ferrule’s connector side	Cement drops on the exposed connector end	Protect each ferrule with a protective sleeve during headcap construction; if cement gets on the ferrule’s connector’s side, wipe it off straight away with an optical cleaning wipe.
Headcap assembly	Implant loosens or detaches within days to weeks	Wet, greasy skull surface; left in place soft tissues; weak anchoring	Remove the periosteum completely, degrease and dry the bone thoroughly, apply tissue adhesive; insert an additional anchoring screw.
Postoperative period	Wound dehiscence around the cap	Rough headcap edge contacting the skin; scratching of the implant by the animal	Keep the cement edge smooth; follow the analgesia regimen; put the protective collar back on the animal for 2–3 days; house animals with protective enrichment (e.g., areas of low light within the cage, non-tangling nesting material, etc.).
Recording	Weak fluorescence signal despite high expression	Fiber tip is too far from the region expressing the biosensor	Confirm tip placement histologically.
